# Changes in Vertebral Marrow Fat Fraction Using 3D Fat Analysis & Calculation Technique Imaging Sequence in Aromatase Inhibitor-Treated Breast Cancer Women

**DOI:** 10.3389/fendo.2022.931231

**Published:** 2022-06-23

**Authors:** Taihu Wan, Yuhang Zhu, Qinghe Han, Lin Liu

**Affiliations:** ^1^ Department of Radiology, China-Japan Union Hospital of Jilin University, Changchun, China; ^2^ Radiology of Department, The Second Hospital of Jilin University, Changchun, China

**Keywords:** breast cancer, aromatase inhibitor, bone mineral density, chemical shift encoding-based, marrow fat, proton density fat fraction

## Abstract

Aromatase inhibitor (AI) is a cornerstone drug for postmenopausal women with estrogen receptor-positive early-stage breast cancer. Fat-bone interactions within the bone marrow milieu are growing areas of scientific interest. Although AI treatment could lead to deterioration of the skeleton, the association between AI medication and subsequent marrow adiposity remains elusive. A total of 40 postmenopausal, early-staged, and hormone receptor-positive breast cancer patients who underwent treatment with adjuvant AIs and 40 matched controls were included. Marrow proton density fat fraction (PDFF) at the L1−L4 vertebral bodies using 3D Fat Analysis & Calculation Technique imaging (FACT) sequence at 3.0T, bone mineral density (BMD) by dual-energy X-ray absorptiometry, and serum bone turnover biomarkers were determined at baseline and at 6 and 12 months. We found that, in comparison to baseline, an increase of type I collagen cross-linked telopeptide was detected at 12 months (*P <*0.05). From baseline to 12 months, the PDFF measured using FACT was greatly increased. At 12 months, the median percent change of PDFF (4.9% vs. 0.9%, *P <*0.05) was significantly different between the AI treatments and controls. The same trend was observed for the marrow PDFF at 6 months relative to the respective values at baseline. Although BMD values were significantly reduced after 12 months in AI-treated women, changes in BMD vs. baseline condition were not significantly different between the AI-treated and control groups [Δ BMD −1.6% to −1.8% vs. −0.3% to −0.6%, respectively, *P* > 0.05]. In the AI-treated group, Δ PDFF was associated with Δ BMD at the lumbar spine (*r* = −0.585, *P* < 0.001), but not in the controls. Taken together, over a 12-month period, spinal marrow fat content assessed with FACT sequence significantly increased in postmenopausal women with hormone-receptor-positive breast cancer receiving AI treatment.

## Introduction

Aromatase inhibitors (AIs) are widely recommended for use by postmenopausal women who have estrogen receptor-positive early-stage breast cancer. Treatment with AIs provides benefits to breast cancer patients in terms of improved disease-free survival and overall survival (1). However, AI-induced deterioration of bone loss and its management with bisphosphonates is still unclear. In addition, the optimal duration of AI therapy for early breast cancer remains elusive.

Extended use of adjuvant endocrine therapy and persistent deterioration of the skeleton from recent findings emphasized the need to assess bone loss and fracture risk in women with hormone-receptor-positive, early-stage breast cancer initiated on AIs (1, 2). Bone mineral density (BMD) evaluation by dual-energy X-ray absorptiometry (DXA) is actually limited. Accuracy of DXA measurements is influenced by degenerative changes in the spine or aortic mineralization and by the variable proportion of fat in overlying soft tissue since it uses a two-dimensional projectional measurement (3). The use of bone quality assessment by means of a based-DXA trabecular bone score may contribute to identifying those with a higher risk of fracture independent of bone density (4, 5). The use of other imaging techniques, such as high resolution peripheral quantitative computed tomography by capturing more and different information on the properties of bone microstructure, have potential implications for clinical practice in the future (6).

Adipocytes in the bone marrow are highly plastic, and have a distinctive characteristic to secrete an extensive number of cytokines and adipokines such as resistin, leptin, and C-C Motif Chemokine Ligand 2 (CCL2) that exert local and endocrine functions. Additionally, bone marrow adipose tissue has been proposed to have mixed brown and white fat characteristics (7, 8). Both animal and human data supported a clinical association between marrow adipose tissue content and integrity of skeleton (9, 10). The proton density fat fraction (PDFF) as a biomarker for osteopenia and osteoporosis enables discrimination of low bone mass from healthy controls (9, 11). Accumulating evidence also highlights the importance of interactions between marrow adipocytes and tumor cells (12, 13). Although a previous study reported that AI-treated patients maintained vertebral marrow PDFF values with a relatively small sample size, prospective changes of marrow fat content in postmenopausal women with breast cancer at completion of AI treatment remain poorly understood.

Therefore, the current study was designed to evaluate the prospective changes in spinal marrow fat content and bone mass in postmenopausal women with early-staged breast cancer after completing AI treatment using chemical shift encoding–based water-fat magnetic resonance imaging (MRI) at 3.0T.

## Materials and Methods

### Participants

This study was performed in accordance with the ethical standards described in the 1964 Declaration of Helsinki and its later amendments. This study was approved by the Institutional Review Board of China-Japan Union Hospital of Jilin University, and all participants provided informed consent. In this prospective, observational study, we recruited 40 postmenopausal women (age, 51.7-73years) with hormone-receptor-positive early-staged breast cancer (including carcinoma *in situ* and stage I−II breast cancers) who were scheduled to receive treatment with adjuvant AIs (i.e., letrozole, anastrozole, and exemestane) between May 2018 and January 2022. Participants were excluded if they had (1): history of lumbar spinal surgery, known or suspected bone metastases, irradiation and/or chemotherapy, other malignancies, distant metastasis, chronic diseases such as rheumatoid arthritis, diabetes mellitus, liver and kidney dysfunction, severe cardiac, hematological, psycho, and nervous system diseases; 2) use of medications known to interfere with fat/bone metabolism such as glucocorticoids, bisphosphonates, denosumab, teriparatide, strontium ranelate, anticoagulants, anticonvulsants, alcohol abuse; (3) bone mineral density or other missing data. A healthy control group (n = 40; age, 51.5-74years) of age-matched postmenopausal women was also recruited from the community.

**Figure 1:**
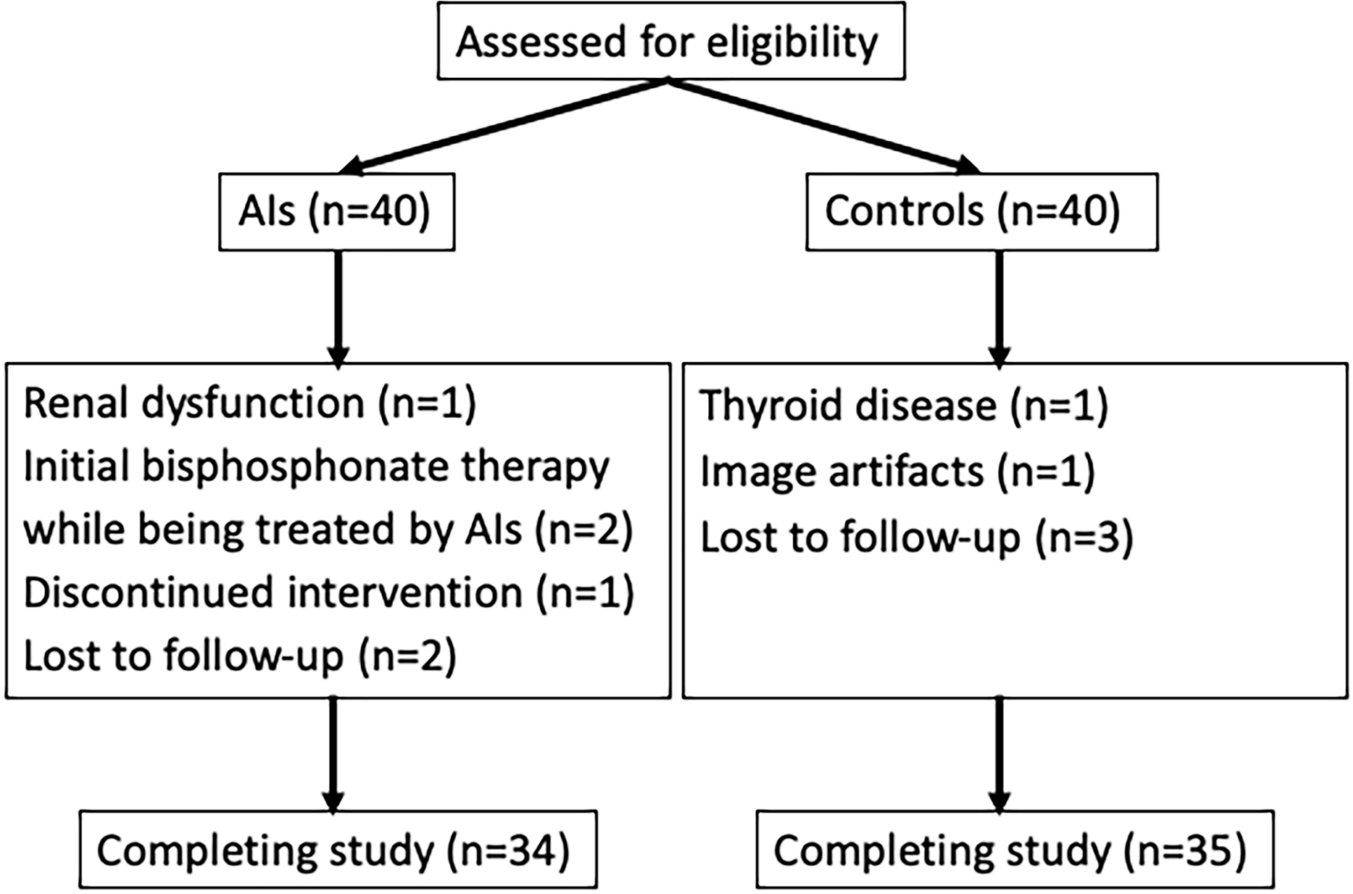
Flow diagram of study subjects. AIs, aromatase inhibitors.

At enrollment, all participants completed self-administered questionnaires about demographics, medical history, general risk factors, family history of breast cancer as well as lifestyle factors (e.g., alcohol consumption, current tobacco smoking, and physical activity). Physical activity was assessed with the International Physical Activity Questionnaire short form, with data reported as Metabolic Equivalent of Task hours per week (14). According to standard procedures, body weight and height were measured at baseline. Body mass index (BMI) was calculated as the weight in kilograms divided by the square of the height in meters. All participants were scheduled to undergo chemical shift encoding-based water-fat MRI, DXA, and serum bone turnover marker analysis at baseline condition, and at 6 and 12 months after receiving endocrine therapy. The study flow chart is presented in **Figure 1**.

### Biochemical Evaluation

Fasting blood samples were collected after overnight fasting and between 7 a.m. and 9 a.m. on the DXA evaluation day. Biochemical evaluation included 25-hydroxyvitamin D, type I collagen cross-linked telopeptide (CTX-I), N-terminal propeptide of type 1 procollagen (P1NP) and osteocalcin. 25-hydroxyvitamin D was measured by immunoassay. Serum CTX-I, P1NP, and osteocalcin were measured by chemiluminescence (ECLIA) in the analyzer Tesmi-F3999 (Tellgen Super Multiplex Immunoassay System, Shanghai, China).

### MRI Acquisition and Analyses

MRI of the lumbar spine was performed on a 3.0 T full-body MRI unit (uMR 780, United Imaging Healthcare, Shanghai, China) to quantify marrow proton density fat fraction (PDFF) at the L1-4 vertebral bodies. Subjects were positioned head-first in the magnet bore in a prone position. Standard clinical MRI protocols, including T1-weighted imaging and T2-weighted imaging (sagittal acquisition), were performed with a built-in 12-channel posterior coil.

**Figure 2:**
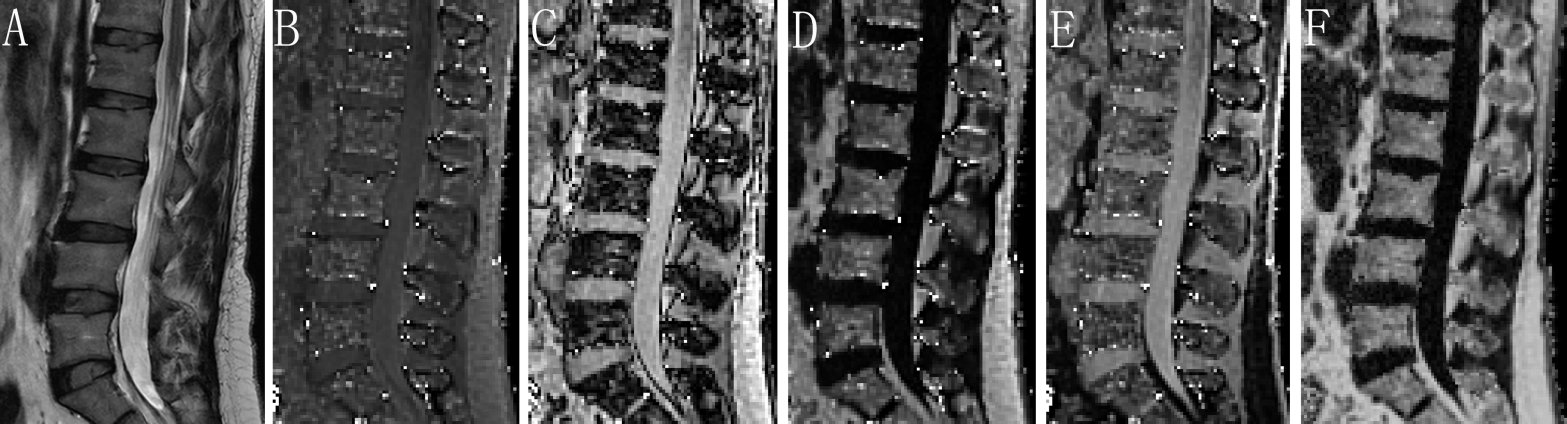
Assessment of PDFF derived from six-echo FACT sequence in image set of a healthy woman. Sagittal T2-weighted **(A)**, in-phase **(B)**, out-of-phase **(C)**, fat-only **(D)**, water-only **(E)**, and the corresponding PDFF map **(F)** with a mean PDFF of 53% at the L1-L4 levels. FACT, Fat Analysis & Calculation Technique imaging; PDFF, proton density fat fraction.

For chemical shift encoding-based water-fat separation at the level of the lumbar spine, a sagittal prescribed 3D Fat Analysis & Calculation Technique (FACT) sequence allowing PDFF quantification, was then acquired with the following parameters: TR= 7.2 ms; six echoes with TE1/ΔTE = 1.21/1.1 ms; flip angle, 3° (low spin flip angle excitation to minimize T1 saturation) (11, 15); slice thickness, 3 mm; interslice gap, 0 mm; acquisition matrix size, 256 × 192; field of view, 380 × 380 mm; 1 average; scan time, 17 seconds. FACT sequence images were transferred to a commercially available workstation (uWS-MR Advanced Postprocess Workstation, United Imaging Healthcare, Shanghai, China). One musculoskeletal radiologist with 5 years’ experience quantitatively analyzed PDFF mappings obtained with FACT sequence (**Figure 2**). The coefficient of variation was 3.07% for the repeatability of PDFF measurement.

### Evaluation of BMD

Areal BMD values at the lumbar spine (L1-L4), femoral neck, and total hip were assessed using dual energy X-ray absorptiometry (DXA, Hologic Discovery). DXA scans were performed by a certified operator. Precision coefficients were 1.17% for the femoral neck, 1.09% for the total hip, and 1.29% for the lumbar spine. Both MRI and DXA examinations were performed on the same day.

### Statistical Analysis

The sample size calculation was performed using G*Power software v3.1, taking into consideration the effect of aromatase inhibitor on fat fraction percentage (16). The effect size of 0.60 showed that with a significance level of 95% and statistical power of 80% (power 1−β = 0.80), the minimum number of participants required was 24. Data are presented as mean ± standard deviation (SD), median (interquartile range, IQR) or n (%) as appropriate. Normality was evaluated by the Shapiro-Wilk test. Student’s *t-test* or Mann-Whitney test was performed to compare quantitative variables and Fisher’s exact or chi-square test for qualitative analyses between groups. The marrow MRI PDFF, DXA BMD, and serum levels of bone turnover biomarkers at baseline and at 6 and 12 months were assessed using the paired *t* test or Wilcoxon rank-sum test. Statistical analyses were performed using SPSS software version 20.0 for Windows (SPSS Inc, Chicago, IL, USA). All statistical tests were two sided, and significance was set at *P <*0.05.

## Results

### Baseline Characteristics of Study Population

A total of 34 postmenopausal women with early breast cancer receiving AI treatment and 35 healthy controls completed the study. As shown in **Figure 1**, 11 participants were excluded from the final analysis: two participants because of initial bisphosphonate therapy while being treated by AIs, two with renal dysfunction and thyroid disease, six with discontinued intervention or lost to follow-up, and the other one because of image artifacts. Over a 12-month period, none of the patients reported any new skeletal-related events. The demographic and clinical characteristics of all participants are shown in **Table 1**. At baseline, no significant differences except for marrow PDFF were observed between the breast cancer women treated with AIs and control groups. Breast cancer patients had higher marrow PDFF than that of the controls.

**Table 1:** Baseline characteristics of the study population.

	AIs (n = 34)	Controls (n = 35)
Age, years	59.2 ± 5.2	59.4 ± 5.8
Time since menopause, years	6.0 (4, 9)	6.5 (4.5, 8.5)
Height, cm	158.8 ± 6.1	159.5 ± 7.0
Weight, kg	60.7 ± 7.1	61.6 ± 7.7
BMI, kg/m^2^	24.1 ± 3.1	24.4 ± 4.1
Alcohol intake, n (%)	1 (2.9)	2 (5.7)
Smokers, n (%)	2 (5.9)	2 (5.7)
5-hydroxyvitamin D, ng/mL	46.1 (33.5, 67.2)	44.8 (30.2, 63.5)
CTX-I, pg/mL	242 (146, 337)	226 (138, 320)
P1NP, ng/mL	39.6 (29.7, 54.8)	41.0 (30.1, 56.4)
Osteocalcin, ng/mL	15.8 ± 4.9	16.5 ± 5.4
Lumbar spine BMD, g/cm^2^	1.052 ± 0.012	1.058 ± 0.009
Total hip BMD, g/cm^2^	0.961 ± 0.011	0.958 ± 0.008
Femur neck BMD, g/cm^2^	0.877 ± 0.007	0.875 ± 0.008
Spinal PDFF, %	59.0 ± 6.3	53.4 ± 5.9 ^a^

Data are presented as mean ± SD, median (IQR) or n (%) as appropriate.

AIs, aromatase inhibitors; BMD bone mineral density; BMI, body mass index; CTX-I, type I collagen cross-linked telopeptide; IQR, interquartile range Q1-Q3; P1NP, N-terminal propeptide of type 1 procollagen; PDFF, proton density fat fraction; SD, standard deviation.

^a^P ^<^0.05 by independent-sample t-test.

### Changes in Marrow PDFF and BMD

**Figure 3:**
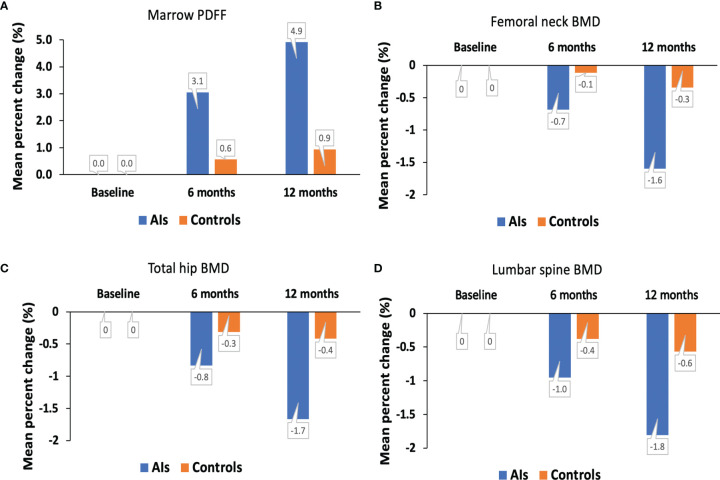
Mean percent change in marrow PDFF **(A)**, BMD at the femoral neck **(B)**, total hip **(C)** and lumbar spine **(D)** over time. AIs, aromatase inhibitors; BMD, bone mineral density; PDFF, proton density fat fraction.

The spinal marrow PDFF, BMD values at the femoral neck, total hip, and lumbar spine from the hormone-receptor-positive early breast cancer patients receiving AI treatment and healthy controls at baseline condition and at 6 and 12 months are shown in **Figure 3**. For the AIs and control groups, changes in marrow PDFF and BMD are shown in **Table 2**. Marrow PDFF at the 6-month follow-up visit (60.8 ± 5.5%) increased significantly compared to PDFF at the initial visit (59.0 ± 6.3%, *P* < 0.001) in the breast cancer patients receiving AIs, but not in the controls (53.7 ± 5.3% vs 53.4 ± 5.9%, *P >*0.05). Relative to the respective values at baseline, the marrow PDFF value at 6 and 12 months markedly increased by a median of 3.1% and 4.9% (all *P <*0.001) in the AIs group, respectively, but not in the controls (0.6% and 0.9%, all *P >*0.05, respectively),

**Table 2:** Changes in bone turnover biomarkers, BMD and spinal marrow PDFF in AIs-treated group.

Parameters	Groups	At baseline	At 6 months	At 12 months	% change	
					Δ_6-0m_	Δ_12-0m_
5-hydroxyvitamin D, ng/mL	AIs	46.1(33.5, 67.2)	45.1(29.8, 63.6)	47.7(34.9, 70.1)	−2.2	3.5
	Controls	44.8 (30.2, 63.5)	45.4 (32.0, 68.1)	47.0 (30.0, 70.4)	1.3	4.9
CTX-I, pg/mL	AIs	242 (146, 337)	260 (151, 355)	291 (176, 378)	7.4	20.2 ^a^
	Controls	226 (138, 320)	231 (144, 331)	238 (152, 353)	2.2	5.3
P1NP, ng/mL	AIs	39.6 (29.7, 54.8)	40.8 (31.5, 57.3)	42.1 (32.0, 60.9)	3.0	6.3
	Controls	41.0 (30.1, 56.4)	42.0 (28.6, 58.9)	40.1 (29.7, 60,1)	2.4	−2.2
Osteocalcin, ng/mL	AIs	15.8 ± 4.9	15.0 ± 4.5	16.3 ± 5.2	−5.1	3.2
	Controls	16.5 ± 5.4	16.7 ± 5.8	16.0 ± 5.7	1.2	−3.0
Lumbar spine BMD, g/cm^2^	AIs	1.052 ± 0.012	1.042 ± 0.011	1.033 ± 0.014	−1.0	−1.8 ^b^
	Controls	1.058 ± 0.009	1.054 ± 0.012	1.052 ± 0.013	−0.4	−0.6
Total hip BMD, g/cm^2^	AIs	0.961 ± 0.011	0.953 ± 0.010	0.945 ± 0.009	−0.8	−1.7 ^b^
	Controls	0.958 ± 0.008	0.955 ± 0.009	0.954 ± 0.012	−0.3	−0.4
Femur neck BMD, g/cm^2^	AIs	0.877 ± 0.007	0.871 ± 0.008	0.863 ± 0.009	−0.7	−1.6 ^b^
	Controls	0.875 ± 0.008	0.874 ± 0.009	0.872 ± 0.008	−0.1	−0.3
Spinal PDFF, %	AIs	59.0 ± 6.3	60.8 ± 5.5	61.9 ± 6.0	3.1 ^b^	4.9 ^b^
	Controls	53.4 ± 5.9	53.7 ± 5.3	53.9 ± 5.5	0.6	0.9

Data are presented as mean ± SD, median (IQR) or % as appropriate.

AIs, aromatase inhibitors; BMD bone mineral density; CTX-I, type I collagen cross-linked telopeptide; IQR, interquartile range Q1-Q3; M, month; P1NP, N-terminal propeptide of type 1 procollagen; PDFF, proton density fat fraction; SD, standard deviation.

To detect difference between various time points, ^a^P value by Wilcoxon rank-sum test and ^b^P value by paired t test (all P <0.05).

In the breast cancer patients receiving AIs, femoral neck BMD (0.863 ± 0.009 g/cm^2^ vs. 0.877 ± 0.007 g/cm^2^), total hip BMD (0.945 ± 0.009 g/cm^2^ vs. 0.961 ± 0.011g/cm^2^), and lumbar spine BMD (1.033 ± 0.014 g/cm^2^ vs. 1.052 ± 0.012 g/cm^2^, all *P <*0.05) were decreased at the 12month follow-up visit compared to the initial visit. In contrast, no significant difference was found in the DXA BMD values at the femoral neck, total hip, and lumbar spine, with a median of –0.7%, −0.8%, and −1.0% (all *P >*0.05), respectively, between baseline condition and at 6 months.

### Changes in Serum Biomarkers

At baseline condition, serum biomarkers including 25(OH)D, CTX-I, P1NP, and osteocalcin levels were not significantly different in the breast cancer patients treated with AIs compared with the controls (**Table 1**). Similar results were observed at 6 months. CTX-I level was significantly increased after 12 months in comparison to baseline values in the AI-treated group, and significant differences were found between the AIs and control groups at 12 months (**Table 2**). No significant differences in the 25(OH)D, P1NP, and osteocalcin levels were observed at different timepoints.

### Relationships Among Marrow PDFF, BMD, and Serum Biomarkers

In the breast cancer patients receiving AIs group, a significantly negative relationship was found between change of marrow PDFF and change of lumbar spine BMD values (*r* = −0.585, *P* < 0.001) at 12 months relative to the respective values at baseline, but not in the controls group. Spinal marrow PDFF variation over time was not significantly related with changes of BMD at the femoral neck and total hip in both the AI-treated breast cancer patients and healthy controls. In the AIs group, Δ bone turnover biomarkers at 6 months and 12 months versus baseline condition was not associated with Δ spinal marrow PDFF or Δ BMD at the femoral neck, total hip, and lumbar spine.

## Discussion

In this prospective observational study, we performed DXA scans, MR FACT sequence, and serological tests to clarify changes in spinal marrow PDFF, BMD at the femoral neck, total hip and lumbar spine, and bone turnover biomarker levels in postmenopausal women with estrogen receptor-positive early-stage breast cancer receiving AIs. We found that vertebral marrow PDFF was significantly increased at 6 and 12 months post-AI treatment onset. We also showed that BMD values at the total hip, femoral neck, and lumbar spine were decreased at the 12-month follow-up visit compared to the initial visit. Changes in marrow PDFF and Δ lumbar spine BMD values were negatively associated in the AIs group.

Bone marrow adipose tissue is now recognized as an endocrine organ. Accumulating evidence indicates that bone marrow fat plays a complex role in bone health, energy metabolism, and hematological diseases like aplastic anemia, multiple myeloma, and leukemia (9, 17). A previous study demonstrated that breast cancer patients had higher marrow fat content in comparison with the age-matched controls. Expansion of marrow fat may be an independent risk factor for postmenopausal breast cancer and clinicopathological characteristics of breast cancer (14). In this present work, as compared with the healthy controls, the hormone-receptor-positive early breast cancer patients receiving AIs showed fat expansion within the bone marrow.

The level of serum β-CTX is used as the reference marker for bone resorption, and P1NP can be measured as one of bone formation biomarkers. During bone formation as well as bone resorption, osteocalcin can be released into the circulation. Several studies indicated that P1NP and β-CTX are the most efficient biomarkers to predict the BMD changes (18). As expected, serum β-CTX markedly elevated at 12 months after AI treatment. Similar to our results, Catalano et al. found that β-CTX levels increased significantly after 9 and 18 months in comparison to baseline values in the AI-treated group (19, 20). In contrast to ours and other studies (19), no significant change was found in serum β-CTX from baseline condition to 12 months in postmenopausal women with early breast cancer at lower and moderate risk of fragility fracture who received AIs (21).

AIs are in widespread use for hormone-receptor-positive breast cancer patients. Several clinical trials have reported AI-related bone loss and fracture risk in both premenopausal and postmenopausal women (4 19, 20, 22). In clinical practice, BMD was used to assess bone strength and risk of fracture. However, in some pathologic conditions such as diabetes mellitus patients, there is an apparent contradiction of elevated bone mass associated with a higher fracture (5), which may be due to poor bone quality assessment with BMD measurement. Seeking imaging methods other than BMD to evaluate bone strength and risk of fracture is of important implication, such as marrow fat fraction, an indirect measure of bone quality (23, 24). The use of chemical shift-encoded MRI or magnetic resonance spectroscopy and bone quality by means of PDFF could additionally help to identify those with bone deterioration or higher risk of fracture independent of BMD (11, 24).

Bone marrow fat tissue composition and quantification may play an important role in bone pathophysiology, but has not been thoroughly studied in AI users. A recent study with a relatively small sample size (n = 8) done by Dieckmeyer et al. (16) showed that over a 12-month period, vertebral bone marrow PDFF was increased by 3.1% in subjects receiving AIs, however it was not significant (*P* = 0.52). Additionally, there was no significant association between PDFF and BMD for the AI treatment group at baseline or follow-up. In our current study with a large sample size and including a group of age-matched healthy controls, we observed that over a 12-month period spinal marrow PDFF was significantly increased in postmenopausal women treated with AIs. *Ex vivo*, estradiol may induce osteogenesis and suppress adipogenesis differentiation of bone marrow mesenchymal stromal cells (25). *In vivo*, estradiol deficiency leads to the increase in bone marrow adipocyte size and number, particularly in postmenopausal osteoporotic women (26). Since treatments with AIs decrease already low endogenous postmenopausal estradiol levels, we found that the PDFF at the lumbar spine was increased by a median of 3.1% at 6 months and 4.9% at 12 months (all *P* < 0.05), respectively. Change of marrow PDFF was associated with change of lumbar spine BMD values at 12 months relative to the respective values at baseline. Thus, marrow PDFF assessed with FACT sequence may be used as a useful early response indicator.

We acknowledge that our study has some limitations. First, the sample size was relatively small, which did not allow to analyze the effects of different AIs (i.e., letrozole, anastrozole, and exemestane) on marrow fat content. This was a single-center study which limits the generalizability of our results. Second, many of the AI-treated breast cancer patients are postmenopausal women who not infrequently have history of multidrug use. Possible interactions between different drugs may affect the bone-fat metabolism that could not be specifically excluded. Third, although we examined both the marrow fat content and BMD, we did not explore their relationships with risk of fractures. Finally, the observation period of AI treatment is typically 5 – 10 years (1), evaluating longitudinal effects over a longer period of time may help to further elucidate the longer-term effects of AIs on vertebral marrow PDFF.

In conclusion, over a 12-month period, spinal marrow proton density fat fraction as measured by FACT sequence significantly increased in postmenopausal women with early breast cancer receiving AI treatment. Our results demonstrated that healthcare professionals for postmenopausal women who received AIs must pay attention to marrow fat content measurements during and after hormone-receptor-positive early breast cancer treatment.

## Data Availability Statement

The original contributions presented in the study are included in the article/supplementary materials. Further inquiries can be directed to the corresponding author.

## Ethics Statement

The studies involving human participants were reviewed and approved by The Institutional Review Board of China-Japan Union Hospital of Jilin University. The patients/participants provided their written informed consent to participate in this study.

## Author Contributions

Study design: TW, LL Study conduct: YZ; Data collection: QH; Data analysis: TW, YZ; Data interpretation: TW, QH; Drafting manuscript: TW, YZ, QH, LL. All authors contributed to the article and approved the submitted version.

## Conflict of Interest

The authors declare that the research was conducted in the absence of any commercial or financial relationships that could be construed as a potential conflict of interest.

## Publisher’s Note

All claims expressed in this article are solely those of the authors and do not necessarily represent those of their affiliated organizations, or those of the publisher, the editors and the reviewers. Any product that may be evaluated in this article, or claim that may be made by its manufacturer, is not guaranteed or endorsed by the publisher.

## References

[1] Waqas K Lima Ferreira J Tsourdi E Body JJ Hadji P Zillikens MC. Updated Guidance on the Management of Cancer Treatment-Induced Bone Loss (CTIBL) in Pre- and Postmenopausal Women With Early-Stage Breast Cancer. J Bone Oncol (2021) 28:100355. doi: 10.1016/j.jbo.2021.100355 33948427 PMC8080519

[2] Chen J Zhang X Lu Y Zhang T Ouyang Z Sun Q. Optimal Duration of Endocrine Therapy With Extended Aromatase Inhibitors for Postmenopausal Patients With Hormone Receptor-Positive Breast Cancer: A Meta-Analysis. Breast Cancer (2021) 28:630–43. doi: 10.1007/s12282-020-01196-8 33387283

[3] Tothill P Weir N Loveland J. Errors in Dual-Energy X-Ray Scanning of the Hip Because of Nonuniform Fat Distribution. J Clin Densitom (2014) 17:91–6. doi: 10.1016/j.jocd.2013.02.008 23522983

[4] Catalano A Gaudio A Agostino RM Morabito N Bellone F Lasco A. Trabecular Bone Score and Quantitative Ultrasound Measurements in the Assessment of Bone Health in Breast Cancer Survivors Assuming Aromatase Inhibitors. J Endocrinol Invest (2019) 42:1337–43. doi: 10.1007/s40618-019-01063-0 31127591

[5] Pechmann LM Petterle RR Moreira CA Borba VZC. Osteosarcopenia and Trabecular Bone Score in Patients With Type 2 Diabetes Mellitus. Arch Endocrinol Metab (2021) 65:801–10. doi: 10.20945/2359-3997000000418 PMC10065394 34762788

[6] Shanbhogue VV Brixen K Hansen S. Age- and Sex-Related Changes in Bone Microarchitecture and Estimated Strength. A Three-Year Prospective Study Using HR-pQCT. J Bone Miner Res (2016) 31(8):1541–9. doi: 10.1002/jbmr.2817 26896351

[7] Hardaway AL Herroon MK Rajagurubandara E Podgorski I. Bone Marrow Fat: Linking Adipocyte-Induced Inflammation With Skeletal Metastases. Cancer Metastasis Rev (2014) 33:527–43. doi: 10.1007/s10555-013-9484-y PMC4154371 24398857

[8] Krings A Rahman S Huang S Lu Y Czernik PJ Lecka-Czernik B. Bone Marrow Fat has Brown Adipose Tissue Characteristics, Which are Attenuated With Aging and Diabetes. Bone (2012) 50:546–52. doi: 10.1016/j.bone.2011.06.016 PMC3214232 21723971

[9] Baum T Rohrmeier A Syvari J Diefenbach MN Franz D Dieckmeyer M. Anatomical Variation of Age-Related Changes in Vertebral Bone Marrow Composition Using Chemical Shift Encoding-Based Water-Fat Magnetic Resonance Imaging. Front Endocrinol (Lausanne) (2018) 9:141. doi: 10.3389/fendo.2018.00141 29670577 PMC5893948

[10] Lecka-Czernik B Stechschulte LA Czernik PJ Sherman SB Huang S Krings A. Marrow Adipose Tissue: Skeletal Location, Sexual Dimorphism, and Response to Sex Steroid Deficiency. Front Endocrinol (Lausanne) (2017) 8:188. doi: 10.3389/fendo.2017.00188 28824548 PMC5543291

[11] Li G Xu Z Gu H Li X Yuan W Chang S. Comparison of Chemical Shift-Encoded Water-Fat MRI and MR Spectroscopy in Quantification of Marrow Fat in Postmenopausal Females. J Magn Reson Imaging (2017) 45:66–73. doi: 10.1002/jmri.25351 27341545

[12] Cha YJ Koo JS. Roles of Omental and Bone Marrow Adipocytes in Tumor Biology. Adipocyte (2019) 8:304–17. doi: 10.1080/21623945.2019.1643189 PMC6768257 31334678

[13] Mukherjee A Chiang CY Daifotis HA Nieman KM Fahrmann JF Lastra RR. Adipocyte-Induced FABP4 Expression in Ovarian Cancer Cells Promotes Metastasis and Mediates Carboplatin Resistance. Cancer Res (2020) 80:1748–61. doi: 10.1158/0008-5472.CAN-19-1999 PMC10656748 32054768

[14] Li G Xu Z Zhuang A Chang S Hou L Chen Y. Magnetic Resonance Spectroscopy-Detected Change in Marrow Adiposity is Strongly Correlated to Postmenopausal Breast Cancer Risk. Clin Breast Cancer (2017) 17:239–44. doi: 10.1016/j.clbc.2017.01.004 28188108

[15] Karampinos DC Ruschke S Dieckmeyer M Eggers H Kooijman H Rummeny EJ. Modeling of T2 * Decay in Vertebral Bone Marrow Fat Quantification. NMR BioMed (2015) 28:1535–42. doi: 10.1002/nbm.3420 26423583

[16] Dieckmeyer M Ruschke S Rohrmeier A Syväri J Einspieler I Seifert-Klauss V. Vertebral Bone Marrow Fat Fraction Changes in Postmenopausal Women With Breast Cancer Receiving Combined Aromatase Inhibitor and Bisphosphonate Therapy. BMC Musculoskelet Disord (2019) 20:515. doi: 10.1186/s12891-019-2916-2 31694630 PMC6836649

[17] Fraczak E Olbromski M Piotrowska A Glatzel-Plucinska N Dziegiel P Dybko J. Bone Marrow Adipocytes in Haematological Malignancies. Acta Histochem (2018) 120:22–7. doi: 10.1016/j.acthis.2017.10.010 29146005

[18] Botella S Restituto P Monreal I Colina I Calleja A Varo N. Traditional and Novel Bone Remodeling Markers in Premenopausal and Postmenopausal Women. J Clin Endocrinol Metab (2013) 98:E1740–8. doi: 10.1210/jc.2013-2264 24001743

[19] Catalano A Morabito N Agostino RM Basile G Gaudio A Atteritano M. Bone Health Assessment by Quantitative Ultrasound and Dual-Energy X-Ray Absorptiometry in Postmenopausal Women With Breast Cancer Receiving Aromatase Inhibitors. Menopause (2017) 24:85–91. doi: 10.1097/GME.0000000000000722 27575547

[20] Catalano A Gaudio A Morabito N Basile G Agostino RM Xourafa A. Quantitative Ultrasound and DXA Measurements in Aromatase Inhibitor-Treated Breast Cancer Women Receiving Denosumab. J Endocrinol Invest (2017) 40:851–7. doi: 10.1007/s40618-016-0606-6 28332172

[21] Van Poznak C Hannon RA Mackey JR Campone M Apffelstaedt JP Clack G. Prevention of Aromatase Inhibitor-Induced Bone Loss Using Risedronate: The SABRE Trial. J Clin Oncol (2010) 28:967–75. doi: 10.1200/JCO.2009.24.5902 20065185

[22] Kuba S Watanabe K Chiba K Matsumoto M Yamanouchi K Fukushima A. Adjuvant Endocrine Therapy Effects on Bone Mineral Density and Microstructure in Women With Breast Cancer. J Bone Miner Metab (2021) 39:1031–40. doi: 10.1007/s00774-021-01239-w 34191126

[23] Patsch JM Li X Baum T Yap SP Karampinos DC Schwartz AV. Bone Marrow Fat Composition as a Novel Imaging Biomarker in Postmenopausal Women With Prevalent Fragility Fractures. J Bone Miner Res (2013) 28:1721–8. doi: 10.1002/jbmr.1950 PMC3720702 23558967

[24] Karampinos DC Ruschke S Gordijenko O Grande Garcia E Kooijman H Burgkart R. Association of MRS-Based Vertebral Bone Marrow Fat Fraction With Bone Strength in a Human *In Vitro* Model. J Osteoporos (2015) 2015:152349. doi: 10.1155/2015/152349 25969766 PMC4417596

[25] Niada S Giannasi C Ferreira LM Milani A Arrigoni E Brini AT. 17beta-Estradiol Differently Affects Osteogenic Differentiation of Mesenchymal Stem/Stromal Cells From Adipose Tissue and Bone Marrow. Differentiation (2016) 92(5):291–7. doi: 10.1016/j.diff.2016.04.001 27087652

[26] Syed FA Oursler MJ Hefferanm TE Peterson JM Riggs BL Khosla S. Effects of Estrogen Therapy on Bone Marrow Adipocytes in Postmenopausal Osteoporotic Women. Osteoporos Int (2008) 19:1323–30. doi: 10.1007/s00198-008-0574-6 PMC2652842 18274695

